# Spleen Stiffness Predicts Survival after Transjugular Intrahepatic Portosystemic Shunt in Cirrhotic Patients

**DOI:** 10.1155/2020/3860390

**Published:** 2020-11-13

**Authors:** Hao Zhu, Huiwen Guo, Xiaochun Yin, Jian Yang, Qin Yin, Jiangqiang Xiao, Yi Wang, Ming Zhang, Hao Han, Yuzheng Zhuge, Feng Zhang

**Affiliations:** ^1^Department of Gastroenterology, Affiliated Drum Tower Hospital, Medical School of Nanjing University, Nanjing, Jiangsu, China; ^2^Department of Gastroenterology, Affiliated Drum Tower Clinical Medical School of Nanjing Medical University, Nanjing, Jiangsu, China; ^3^Department of Ultrasound, Affiliated Drum Tower Hospital, Medical School of Nanjing University, Nanjing, Jiangsu, China

## Abstract

**Objectives:**

Transjugular intrahepatic portosystemic shunt (TIPS) is an effective intervention for portal hypertensive complications. Little is known about the ability of spleen stiffness (SS) for predicting the survival of cirrhotic patients undergoing TIPS. This study is to evaluate the influence of SS detected by point shear wave elastography (pSWE) in predicting survival after TIPS.

**Methods:**

This retrospective cohort study screened consecutive patients who underwent TIPS and reliable pSWE measurement between October 2014 and September 2017 from our prospectively maintained database. SS values were measured before TIPS. The primary endpoint was the overall survival after TIPS. The Cox regression analysis model was used for univariate and multivariate analyses. A receiver operating characteristic (ROC) curve analysis was performed to calculate the sensitivity, specificity, and positive and negative predictive values.

**Results:**

A total of 89 patients were involved in the final analysis. 24 patients (27.0%) died during a median follow-up time of 31 m. Multivariable Cox regression analysis confirmed that higher SS value (*P* < 0.001), LS value (*P* = 0.008), diameter of shunt (*P* = 0.001), and older age (*P* < 0.001) were independent prognostic factors of survival after TIPS. The risk of death rose 57.440-fold for each SS unit (m/s) increase. SS was also correlated with liver failure after TIPS. ROC analysis showed that the best SS cutoff value was 3.60 m/s for predicting survival, with a sensitivity of 54.2% and specificity of 90.8%.

**Conclusions:**

The SS value determined by pSWE in cirrhotic patients was an independent predictive factor for survival after TIPS.

## 1. Introduction

Portal hypertension caused by liver cirrhosis often leads to gastroesophageal variceal bleeding and ascites, which remains a challenging task in clinical practice. For years, transjugular intrahepatic portosystemic shunt (TIPS) has been an effective procedure to manage variceal bleeding and refractory ascites by reducing the portal pressure in patients with advanced cirrhosis. However, TIPS is often associated with a relatively high risk of postprocedure complications, such as liver failure and hepatic encephalopathy, and sometimes with high mortality [1]. Different clinical characteristics in patients often result in a significant difference in prognosis after TIPS; that is, some patients cannot benefit from TIPS in survival [2]. Therefore, it is important to identify suitable patients for TIPS to improve survival.

Liver stiffness (LS) and spleen stiffness (SS) have been proved to be correlated with hepatic venous pressure gradient in compensated and decompensated cirrhotic patients by many studies [3–6], while few works are aimed at demonstrating the association between LS/SS and survival, especially in patients undergoing TIPS [7]. Lee et al. demonstrated that LS detected using MRE was a predictive factor for the development of decompensation and overall survival in cCLD patients [8]. To the best of our knowledge, only one study revealed that increased LS after TIPS was a predictor of organ failure and death; however, the change of LS after TIPS could not be used to guide the selection of proper patients before the procedure and the effect of SS was not evaluated [9]. In addition, although this is the largest cohort of patients with TIPS, the sample size is still rather small and LS measurement is performed using different ultrasound techniques. Recently, Takuma et al. for the first time demonstrated that SS tested by ARFI imaging could predict survival and hepatic decompensation of cirrhotic patients, which might be a useful noninvasive test to predict patient outcomes [10]. However, the predicting power of SS in cirrhotic patients undergoing TIPS has not been revealed so far. Of note, most previous studies evaluated the SS value using transient elastography (TE), but no study has shown the SS value detected by point shear wave elastography (pSWE) to be a predictor of outcome.

The aim of the present study was to evaluate SS detected by pSWE in predicting survival after TIPS in cirrhotic patients.

## 2. Patients and Methods

### 2.1. Patients

This retrospective observational cohort study was conducted in the Department of Gastroenterology in the Affiliated Drum Tower Hospital of Nanjing University between October 2014 and September 2017. Cirrhotic patients aged 18 to 80 years old with successful TIPS insertion and pSWE measurement of the liver and spleen before TIPS were screened and included from our prospectively maintained database of TIPS cohort studies. The exclusion criteria were as follows: severe portal vein thrombosis, concomitant malignant tumors, splenectomy, failure of TIPS procedure, and unreliable pSWE measurement. Written informed consent was obtained from each patient. The whole study was performed following the principles of the 1975 Declaration of Helsinki and was approved by the Ethics Committee of the Affiliated Drum Tower Hospital of Nanjing University.

### 2.2. pSWE Procedure of the Liver and Spleen

pSWE measurement was performed using the techniques described previously [11]. A 4C1 curved array transducer (Acuson S2000, Siemens Medical Solutions) was used to perform both real-time gray-scale imaging and pSWE, which was performed after an overnight fast one day before TIPS procedure. LS of the patients was detected in a supine position. pSWE was used to measure the shear wave velocity (SWV) of the liver using the intercostal approach during real-time gray-scale imaging. The measurements were standardized according to the protocol described previously [11]. Detection of SS was performed using the same methodology. Five valid SWV measurements were tested in the liver or spleen for each patient, and the mean values of SWV were calculated, which were expressed in m/s. Only SWV with an interquartile range ≤ 30% of the median value were considered reliable. The procedures for pSWV measurement were performed by 2 observers with over five years of experience in sonographic examination.

### 2.3. Transjugular Intrahepatic Portosystemic Shunt Procedure

TIPS procedure was performed using the techniques described previously [12]. Briefly, the right or middle hepatic vein was catheterized using a transjugular venous approach. An intrahepatic tract from the hepatic vein to the portal vein was punctured. A 6-8 mm diameter covered stent (Fluency; Bard) or a covered stent combined with a bare metal stent (Luminexx; Bard) was deployed into the tract to support the parenchymal channel. And then the stent was dilated with a balloon catheter. A special catheter with opaque markers was used to identify the stent length. If evident stomach and esophageal varices presented, embolization was performed to fill the residual varices with coils or tissue-adhesive glue.

### 2.4. Follow-Up

Patients' follow-up was performed by the outpatient clinic and telephone calls with an interval of 1-3 months. The follow-up was ended in June 2019. The primary endpoint was death or liver transplantation. The patient would be censored at the time if lost to follow-up or alive until the deadline. Survival time was calculated from the date of the TIPS procedure. Furthermore, the variceal rebleeding, liver failure, shunt dysfunction, and hepatic encephalopathy after TIPS were also investigated.

### 2.5. Statistical Analysis

Data were presented as median and ranges unless otherwise declared. The Mann-Whitney *U* test and Fisher's exact test were used to assess the differences between the two groups. Survival curves were calculated using the Kaplan-Meier method and were compared using the log-rank test. The Cox regression analysis (backward stepwise likelihood quotient) model was used for univariate and multivariate analyses. Variables with *P* value < 0.05 were entered into the multivariate analysis to identify the individual predicting factors. A receiver operating characteristic (ROC) curve analysis was performed to calculate the sensitivity, specificity, and positive and negative predictive values. The SS value with the best specificity and sensitivity (Youden's index) was chosen to optimize the predictive ability of the cutoff values for the overall survival. All statistical analyses were performed using SPSS version 25.0 (SPSS Inc., Chicago, Illinois, USA). Values of *P* less than 0.05 were considered statistically significant.

## 3. Results

### 3.1. Patients' Baseline Characteristics

**Figure 1:**
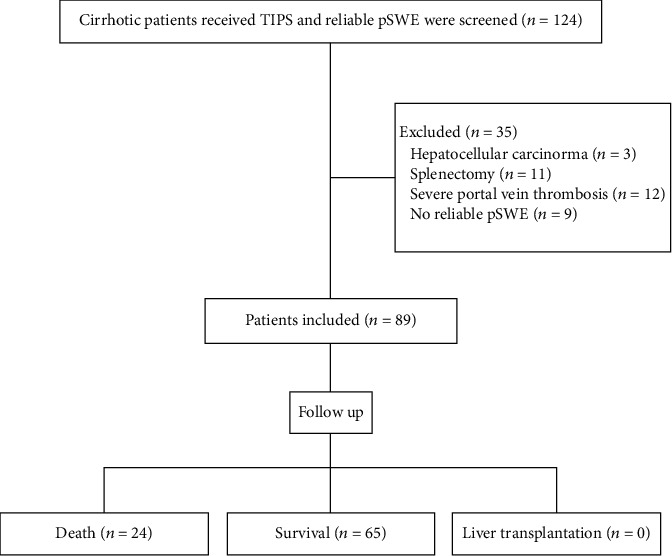
Patient flow chart.

**Figure 2:**
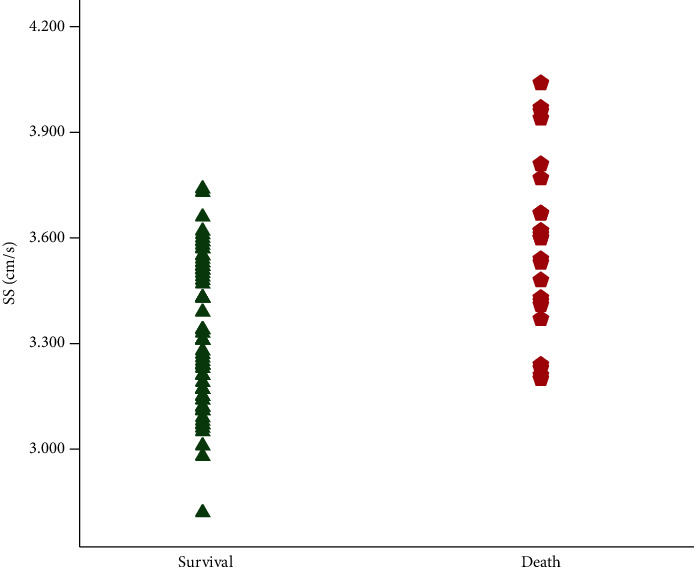
Spleen stiffness (SS) in patients with different prognoses. Each point represents a single patient.

From October 2014 to September 2017, we screened a cohort of 124 cirrhotic patients who received successful TIPS insertion and pSWE measurement of the liver and spleen before TIPS in the Affiliated Drum Tower Hospital of Nanjing University Medical School, regardless of the diagnosis and indication for TIPS. Among these, 35 patients were excluded due to severe portal vein thrombosis, concomitant malignant tumors, splenectomy, or no reliable pSWE measurement. Finally, a total of 89 patients were included in the study (Figure 1). Patients' baseline characteristics are shown in Table 1. The median age was 59 years, ranging from 30 to 80. 54 patients (60.7%) were men. The median LS and SS SMV were 1.96 cm/s and 3.43 cm/s, respectively. The age, CTP score, MELD score, SB, LS, and SS were significantly different between death and survival. The patients with poor survival had a higher value of SS (Figure 2).

### 3.2. Outcome of TIPS and Follow-Up

100% technical success of TIPS was achieved in this cohort of patients. And no patients died from TIPS procedure. The median portal vein pressure (PVP) before TIPS shunt insertion was 29.4 mmHg, ranging from 16.0 to 42.0, which decreased to 21.3 mmHg, from 10.0 to 33.0. The number of patients using a 6/7/8 mm diameter of shunt was 19/21/49. The median follow-up time was 31 m, ranging from 3 to 57 (Table Suppl 1). During the follow-up, 11 (14.5%) patients had variceal rebleeding. 24 patients (27.0%) died as follows: variceal bleeding (*n* = 2), hepatic encephalopathy or liver failure (*n* = 17), liver cancer (*n* = 2), infection including spontaneous bacterial peritonitis (*n* = 3), and non-liver-related death (*n* = 0). No patient received liver transplantation. The cumulative 1-, 2-, and 3-year overall survival rates were 88.6%, 81.8%, and 76.6%, respectively (Figure Suppl 1).

### 3.3. Predicting Factors Associated with Survival after TIPS

Fifteen variables were entered into the univariate Cox regression model including LS and SS. The diameter of shunt, which was different in this cohort of patients, was also involved to evaluate the effect of shunt on survival. The result showed that the age, CTP score, MELD score, serum bilirubin, serum Cr, ascites, diameter of shunt, LS, and SS were significantly correlated with survival (*P* < 0.05) (Table 2). The nine variables were evaluated in the multivariate analysis, which showed that the age, diameter of shunt, LS, and SS were independent prognostic factors of survival. Among these, SS had the strongest predicting power (*P* < 0.001). The risk of death rose 57.440-fold for each SS unit (m/s) increase. In addition, SS, ascites, and MELD score were also correlated with liver failure after TIPS, evaluating by the univariate and multivariate Cox regression model (Table Suppl 2). The risk of liver failure rose 140.755-fold for each SS unit (m/s) increase.

### 3.4. Determination of SS Cutoff Values for Predicting Survival

**Figure 3:**
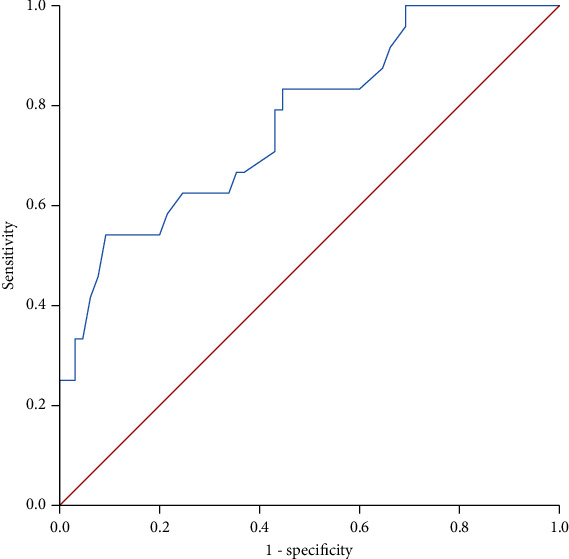
Receiver operating characteristic (ROC) curve of spleen stiffness (SS) in predicting survival after TIPS.

**Figure 4:**
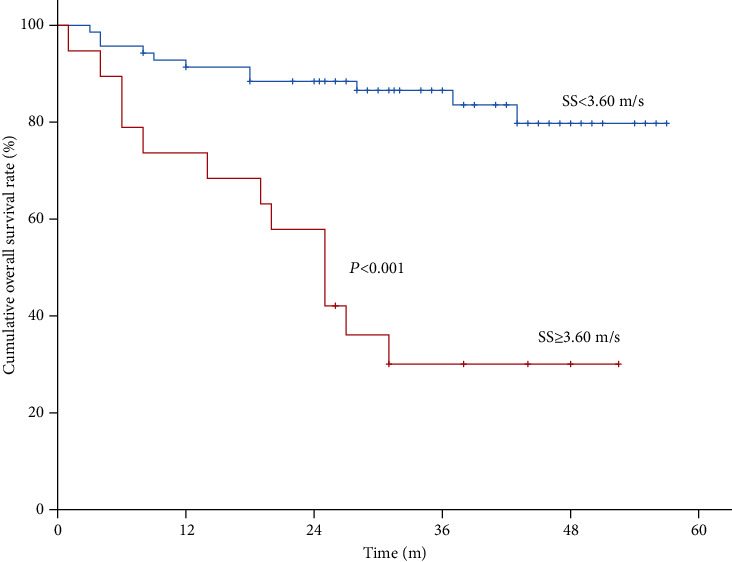
Kaplan-Meier curves of spleen stiffness (SS) in predicting survival after TIPS with a cutoff of 3.60 m/s.

The ROC curve of SS is shown in Figure 3, by which the biggest Youden's index was 0.45 and AUROC was 0.769 (0.657-0.881). The cutoff value of SS was 3.60 m/s, and the sensitivity, specificity, NPV, PPV, and accuracy were 54.2% (13/24), 90.8% (59/65), 84.3% (59/70), 68.4% (13/19), and 80.9% (72/89), respectively. The cumulative overall survival rate was significantly lower for the patients with SS ≥ 3.60m/s than the patients with SS < 3.60m/s (*P* < 0.0001; Figure 4).

## 4. Discussion

In this study, we examined preoperative SS of 89 cirrhotic patients undergoing TIPS procedure. The predicting value of SS in the survival after TIPS was analyzed for the first time. Our result showed that SS was an independent prognostic factor of survival after TIPS (*P* < 0.001). And the best SS cutoff value was 3.60 m/s for predicting survival, with a sensitivity of 54.2% and specificity of 90.8%.

TIPS has been proven to be effective in patients with variceal bleeding, which is superior to endoscopic therapy combined with pharmacotherapy for the prevention of rebleeding. Moreover, TIPS is supported as the first-line intervention in selected patients owing to remarkable survival benefits [13, 14]. A recent study showed that LS immediately decreased after TIPS implantation due to portal pressure changes [15]; however, the outcome was not mentioned. To the best of our knowledge, only one study by Jansen et al. claimed that increased LS after TIPS was a predictor of organ failure and death; however, it was not useful for guiding the selection of proper patients before the procedure [9]; meanwhile, SS was not evaluated in the study. A further limitation is the rather small size of the cohort, and TE was performed in patients with large ascites.

TE has been widely used to evaluate LS and SS [16]; however, there are technical limitations for TE, such as obesity, narrow intercostal spaces, and ascites. EFSUMB guidelines claimed that TE could not be performed in patients with perihepatic ascites [17]. On the other hand, most patients with decompensated cirrhosis had ascites, especially those candidates for TIPS. Therefore, although TE performed well in patients with compensated cirrhosis, there was uncertainty in patients with decompensated cirrhosis. To overcome this limitation, we used pSWE to detect LS and SS. pSWE is a new shear wave-based method that can evaluate the tissue stiffness by measuring the speed propagation of shear waves generated by US [7, 18]. EFSUMB guidelines also demonstrated that pSWE could be utilized in patients with ascites [17].

Many studies showed that LS detected by different methods was correlated with hepatic vein pressure gradient (HVPG) [19]. In addition, in a systematic review involving 17 prospective cohort studies, baseline LS was found to be associated with the risk of death in patients with chronic liver disease; however, the predictive power is relatively low [20]. Meanwhile, SS seemed to be better than LS in predicting portal hypertension and outcome [21–23]. SS could assess changes in portal pressure after liver transplantation and decreased significantly when portal hypertension was resolved [24]. Other studies also showed that SS evaluated by pSWE and ARFI was closely correlated with portal hypertension and HVPG [11, 25, 26]. In 2 systematic reviews, the diagnostic performance of SS in detecting the presence of esophageal varices was significantly better than that of LS [27, 28]. And Sultanik et al. demonstrated that baseline LS measurement could not predict disease progression of HCV patients with cirrhosis [29]. Recently, Takuma et al. for the first time demonstrated that SS tested by ARFI imaging could predict not only death but also hepatic decompensation of cirrhotic patients, which might be a useful noninvasive test to predict patient outcomes [10]. Based on this study, the indication for TIPS in patients with higher SS values should be determined carefully.

A newly published meta-analysis revealed that the correlation between SS and HVPG was good (AUC 0.92), but the different cutoff values and techniques among studies might limit the impact on clinical practice [30]. Buechter et al. demonstrated that SS reflected more accurately the dynamic changes concerning the splanchnic circulation occurring in advanced stages of cirrhosis compared to LS [31]. These results indicated that SS might be related with survival. On the other hand, SS was proved to decrease significantly after TIPS implantation [31]. A small sample prospective study conducted by De Santis et al. demonstrated that SS was superior to LS in detecting the change of portal pressure induced by TIPS. This work makes SS a potential noninvasive method to evaluate portal hypertension. Therefore, further investigations are needed to establish the applicability of SS in the management of portal hypertension [32]. However, no study has revealed the relation between SS and survival after TIPS. Until now, our study for the first time demonstrated that SS evaluated by pSWE could predict survival after TIPS with 80.9% accuracy, while the accuracy by Takuma et al. was 76%.

In this study, we also found that SS was not the only predictor of survival. The age, diameter of shunt, and LS were also correlated with post-TIPS survival, which was reasonable and explicable. However, SS was the most powerful prognostic predictor. To further validate the effect of SS, the cutoff value of SS was detected as 3.60 m/s, which was comparable with the cutoff of 3.53 m/s found by Takuma et al. in patients with decompensated cirrhosis [10]. In addition, we also evaluated the effect of SS on liver failure, which was the first cause of death for patients undergoing TIPS. And the result showed that SS was also a strong predictor of post-TIPS liver failure.

There are several limitations to the present study. Firstly, the sample size is relatively small. However, this has been the largest cohort of patients with TIPS. Secondly, the diameter of TIPS shunt (range 6 to 8 mm) was not unified due to the different selection of doctors according to their experience. Because the diameter of shunt might be related with the outcome and survival after TIPS [33], we involved the parameter in the Cox analysis to evaluate and adjust the influence. Fortunately, the diameter of shunt did not affect our conclusion. The result showed that the diameter of shunt was an individual predicting factor for survival after TIPS (*P* = 0.001). However, SS was a predictor with the strongest power. Our results remain therefore robust. In addition, this study is retrospective, but we obtain the cohort from our prospectively maintained database of TIPS over 10 years, which can ensure the accuracy and integrity of the data.

## 5. Conclusion

In conclusion, the present study, for the first time, demonstrates that the SS value determined by pSWE in cirrhotic patients was an independent predictive factor for survival after TIPS.

## Figures and Tables

**Table 1:** Patient demographics, liver disease characteristics, and clinical presentation (median and ranges).

Variables	Overall population (*n* = 89)	Death (*n* = 24)	Survival (*n* = 65)	*P* value
Age (yr)	59 (30-80)	59 (40-80)	58 (30-78)	0.045
Sex (male/female)	54/35	16/8	38/27	0.626
Etiology of liver cirrhosis (viral/others)	50/39	15/9	35/30	0.631
Indication for TIPS^1^ (bleeding/ascites/both)	73/4/12	17/2/5	56/2/7	0.764
CTP^2^ score	7 (5-10)	8 (6-10)	7 (5-10)	0.019
CTP classification (A/B/C)	24/62/3	4/18/2	20/44/1	0.147
MELD^3^ score	10 (6-21)	10.5 (7-21)	10 (6-16)	0.048
SB^4^ (*μ*mol/L)	16.4 (6.3-78.6)	21.6 (6.8-78.6)	16.1 (6.3-51.3)	0.010
PLT^5^ (×10^12^/L)	55 (16-207)	46.5 (16-116)	55 (22-207)	0.348
PT^6^ (s)	15.0 (11.5-20.1)	15.7 (12.5-20.1)	15.0 (11.5-19.4)	0.114
ALT^7^ (IU/L)	21.0 (5.2-136.7)	22.5 (5.2-136.7)	20.3 (6.0-66.2)	0.857
AST^8^ (IU/L)	26.6 (14.0-159.3)	26.3 (18.8-159.3)	26.7 (14-80.1)	0.524
ALB^9^ (g/L)	33.1 (19.8-40.0)	32.2 (23.6-38.9)	33.5 (19.8-40.0)	0.482
Cr^10^ (*μ*mol/L)	64 (37-269)	70 (47-269)	62 (37-144)	0.157
Mild PVT^11^ (yes/no)	13/76	3/21	10/55	1.000
Ascites (no/mild/moderate/large)	17/36/20/16	2/8/7/7	15/28/13/9	0.148
Liver stiffness (m/s)	1.96 (1.46-3.39)	2.08 (1.65-3.39)	1.92 (1.46-2.99)	0.025
Spleen stiffness (m/s)	3.43 (2.82-4.04)	3.60 (3.20-4.04)	3.31 (2.82-3.74)	<0.001

Data are medians (ranges). ^1^TIPS: transjugular intrahepatic portosystemic shunt; ^2^CTP: Child-Turcotte-Pugh; ^3^MELD: model for end-stage liver disease; ^4^SB: serum bilirubin; ^5^PLT: platelet; ^6^PT: prothrombin time; ^7^ALT: alanine aminotransferase; ^8^AST: aspartate aminotransferase; ^9^ALB: albumin; ^10^Cr: creatinine; ^11^PVT: portal vein thrombosis.

**Table 2:** Univariate and multivariate analyses for predicting factors associated with survival after TIPS.

Variable	Univariate analysis		Multivariate analysis	
	HR (95% CI)	*P* value	HR (95% CI)	*P* value
Age (yr)	1.040 (1.002-1.080)	0.041	1.096 (1.044-1.150)	<0.001
Sex (male)	0.704 (0.301-1.646)	0.418		
CTP^1^ score	1.488 (1.078-2.054)	0.016		
MELD^2^ score	1.252 (1.075-1.459)	0.004		
SB^3^ (*μ*mol/L)	1.028 (1.005-1.050)	0.015		
PLT^4^ (×10^12^/L)	0.994 (0.980-1.007)	0.346		
ALT^5^ (IU/L)	1.011 (0.996-1.027)	0.157		
AST^6^ (IU/L)	1.011 (0.997-1.025)	0.113		
ALB^7^ (g/L)	0.984 (0.888-1.090)	0.754		
Cr^8^ (*μ*mol/L)	1.011 (1.000-1.023)	0.045		
PVT^9^	1.387 (0.413-4.657)	0.597		
Ascites	1.727 (1.149-2.596)	0.009		
Diameter of shunt	2.479 (1.204-5.102)	0.014	3.450 (1.684-7.068)	0.001
Liver stiffness (m/s)	4.575 (1.667-12.560)	0.005	5.038 (1.530-16.585)	0.008
Spleen stiffness (m/s)	29.273 (6.211-137.976)	<0.001	57.440 (10.025-329.115)	<0.001

^1^CTP: Child-Turcotte-Pugh; ^2^MELD: model for end-stage liver disease; ^3^SB: serum bilirubin; ^4^PLT: platelet; ^5^ALT: alanine aminotransferase; ^6^AST: aspartate aminotransferase; ^7^ALB: albumin; ^8^Cr: creatinine; ^9^PVT: portal vein thrombosis.

## Data Availability

All data are contained in the manuscript.

## Abbreviations

TIPS: Transjugular intrahepatic portosystemic shunt
LS: Liver stiffness
SS: Spleen stiffness
TE: Transient elastography
pSWE: Point shear wave elastography
SWV: Shear wave velocity
ROC: Receiver operating characteristic
PVP: Portal vein pressure
CTP: Child-Turcotte-Pugh
HVPG: Hepatic venous pressure gradient.

## Additional Points

*Key Points*. The spleen stiffness value in cirrhotic patients was an independent predictive factor for survival after TIPS. Spleen stiffness predicted liver failure after TIPS.

## Supplementary Materials

Supplementary Materials Table Suppl 1: hemodynamic parameters of patients during TIPS insertion. Table Suppl 2: univariate and multivariate analyses for predicting factors associated with liver failure after TIPS. Figure Suppl 1: the cumulative 1-, 2-, and 3-year overall survival rates of patients.
