# Supplementary material for: Spleen Stiffness Predicts Survival after Transjugular Intrahepatic Portosystemic Shunt in Cirrhotic Patients
Source: Biomed Res Int. 2020 Nov 13;2020:3860390. doi: 10.1155/2020/3860390 (PMC7685811; doi:10.1155/2020/3860390)

**Table suppl 1 hemodynamic parameters of patients during TIPS insertion**

| Variables | Overall population (n=89) | Death (n=24) | Survival (n=65) | P value |
| --- | --- | --- | --- | --- |
| Pre-TIPS^1^ PVP^2^ (mmHg) | 29.4 (16.0-42.0) | 29.8 (22.1-42.0) | 29.4 (16-40) | 0.078 |
| Post-TIPS PVP (mmHg) | 21.3 (10.0-33.0) | 22.1 (10.0-31.0) | 20.6 (11.8-33.0) | 0.577 |
| Diameter of shunt (6/7/8mm) | 19/21/49 | 1/4/19 | 18/17/30 | 0.013 |

Data are medians and (ranges). ^1^TIPS, transjugular intrahepatic portosystemic shunt; ^2^PVP, portal vein pressure.

**Table suppl 2 Univariate and multivariate analysis for predicting factors associated with liver failure after TIPS**

| **Variable** | **Univariate analysis** | | **Multivariate analysis** | |
| --- | --- | --- | --- | --- |
|  | **HR (95%CI)** | **P value** | **HR (95%CI)** | **P value** |
| Age (yr) | 1.022 (0.969-1.077) | 0.430 |  |  |
| Sex (male) | 0.815 (0.265-2.847) | 0.815 |  |  |
| CTP^1^ score | 1.769 (1.090-2.871) | 0.021 |  |  |
| MELD^2^ score | 1.293 (1.031-1.619) | 0.025 | 1.305 (1.016-1.677) | 0.037 |
| SB^3^ (umol/L) | 1.016 (0.976-1.057) | 0.459 |  |  |
| PLT^4^ ( ×10^12^/L) | 0.996 (0.977-1.015) | 0.667 |  |  |
| ALT^5^ (IU/L) | 1.004 (0.977-1.032) | 0.768 |  |  |
| AST^6^ (IU/L) | 1.005 (0.981-1.030) | 0.699 |  |  |
| ALB^7^ (g/L) | 1.016 (0.870-1.188) | 0.838 |  |  |
| Cr^8^ (umol/L) | 0.999 (0.973-1.026) | 0.962 |  |  |
| PVT^9^ | 0.536 (0.069-4.192) | 0.552 |  |  |
| Ascites | 2.922 (1.478-5.775) | 0.002 | 3.328 (1.460-7.582) | 0.004 |
| Diameter of shunt | 2.328 (0.828-6.548) | 0.109 |  |  |
| Liver stiffness (m/s) | 4.383 (0.988-19.444) | 0.052 |  |  |
| Spleen stiffness (m/s) | 131.658 (12.931-1340.447) | <0.001 | 140.755 (9.597-2064.344) | <0.001 |

^1^CTP, Child-Turcotte-Pugh; ^2^MELD, Model for end-stage liver disease; ^3^SB, serum bilirubin; ^4^PLT, platelet; ^5^ALT, alanine aminotransferase; ^6^AST, aspartate aminotransferase; ^7^ALB, albumin; ^8^Cr, creatinine; ^9^PVT, portal vein thrombosis.

**Figure suppl 1 Cumulative overall survival rate of patients**


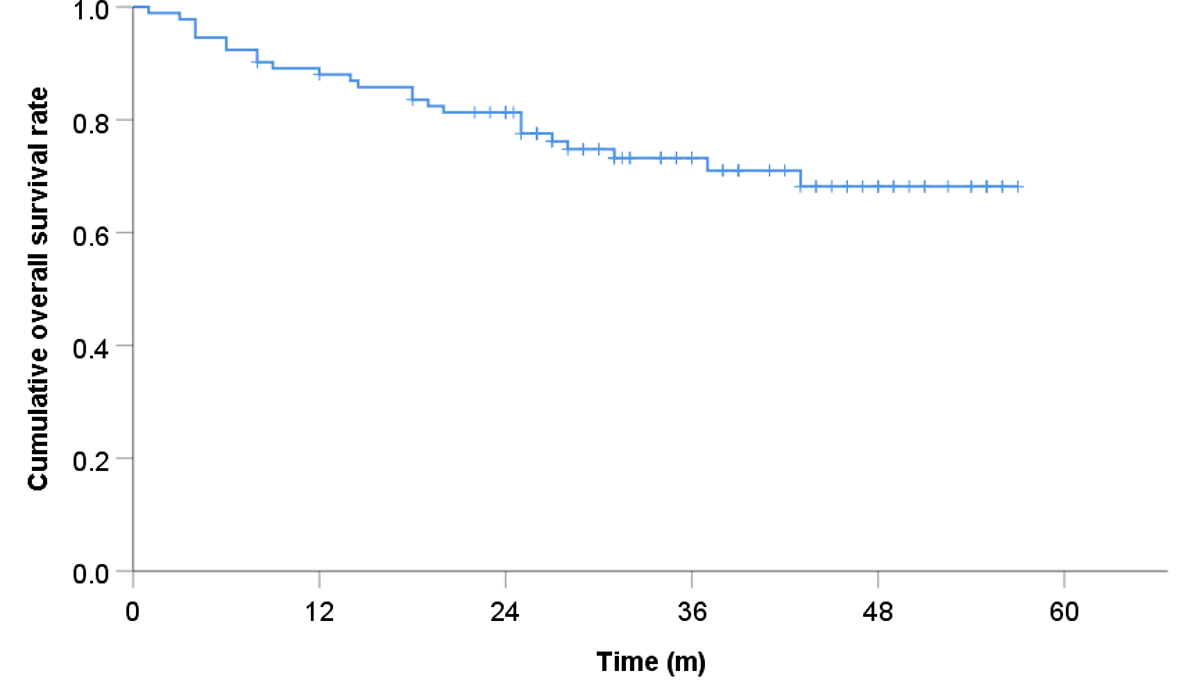

Supplement: Supplementary Materials — Table Suppl 1: hemodynamic parameters of patients during TIPS insertion. Table Suppl 2: univariate and multivariate analyses for predicting factors associated with liver failure after TIPS. Figure Suppl 1: the cumulative 1-, 2-, and 3-year overall survival rates of patients. [file 3860390.f1.docx]
